# Cellular calcium in bipolar disorder: systematic review and meta-analysis

**DOI:** 10.1038/s41380-019-0622-y

**Published:** 2019-12-04

**Authors:** Paul J. Harrison, Nicola Hall, Arne Mould, Noura Al-Juffali, Elizabeth M. Tunbridge

**Affiliations:** 1 Department of Psychiatry, Warneford Hospital, University of Oxford, Oxford, OX3 7JX UK; 2 Oxford Health NHS Foundation Trust, Oxford, UK

**Keywords:** Bipolar disorder, Diagnostic markers

## Abstract

Calcium signalling has long been implicated in bipolar disorder, especially by reports of altered intracellular calcium ion concentrations ([Ca^2+^]). However, the evidence has not been appraised critically. We carried out a systematic review and meta-analysis of studies of cellular calcium indices in bipolar disorder. 2281 records were identified and 117 screened, of which 32 were eligible and 21 were suitable for meta-analyses. The latter each involved up to 642 patients and 404 control subjects. We found that basal free intracellular [Ca^2+^] is increased in bipolar disorder, both in platelets and in lymphocytes. The effect size is 0.55, with an estimated elevation of 29%. It is observed in medication-free patients. It is present in mania and bipolar depression, but data are equivocal for euthymia. Cells from bipolar disorder individuals also show an enhanced [Ca^2+^] response to stimulation with 5-HT or thrombin, by an estimated 25%, with an effect size of 0.63. In studies which included other diagnoses, intracellular basal [Ca^2+^] was higher in bipolar disorder than in unipolar depression, but not significantly different from schizophrenia. Functional parameters of cellular Ca^2+^ (e.g. calcium transients), and neuronal [Ca^2+^], have been much less investigated, and no firm conclusions can be drawn. In summary, there is a robust, medium effect size elevation of basal and stimulated free intracellular [Ca^2+^] in bipolar disorder. The results suggest altered calcium functioning in the disorder, and encourage further investigations into the underlying mechanisms, and the implications for pathophysiology and therapeutics.

## Introduction

The possible involvement of calcium ions ([Ca^2+^]) in bipolar disorder has been under investigation for almost a 100 years [1]. Researchers have compared a range of Ca^2+^ parameters between cases and controls, but following landmark studies in the late 1980s [2, 3], the main focus has been upon intracellular calcium ion concentrations ([Ca^2+^]), predominantly in platelets and lymphocytes. The postulated links between Ca^2+^ and bipolar disorder are usually interpreted in the context of the fundamental roles of Ca^2+^ in neuronal excitation, transmitter synthesis and release, and synaptic function and plasticity [4–8]. Researchers also noted the effects of abnormal Ca^2+^ levels on mood (e.g. in hyperparathyroidism), and the fact that lithium and some other psychotropic drug classes alter Ca^2+^ functioning [9–11].

Over the past decade the study of Ca^2+^ in bipolar disorder has been received new impetus from the increasingly compelling evidence that voltage-gated calcium channels (VGCCs) are part of the genetic aetiology [12, 13]. Interest is enhanced by the therapeutic opportunities which VGCCs provide, given their known druggability by existing calcium channel blockers and the gabapentinoids [14–17]. However, despite multiple studies and several narrative reviews, there is no consensus as to the specific Ca^2+^ abnormality—if any—which characterises bipolar disorder, nor is the robustness of the evidence clear [18–20]. To address this gap, we have systematically reviewed and meta-analysed the literature regarding cellular Ca^2+^ in bipolar disorder.

## Materials and methods

We registered our study on the PROSPERO international prospective register of systematic reviews (http://www.crd.york.ac.uk/PROSPERO/display_record.php?ID=CRD42019119254). The primary question we wished to answer was: Does cellular [Ca^2+^] differ in cells from individuals with bipolar disorder compared with healthy controls, either at baseline or in response to stimulation? Our secondary analyses included comparisons between different cell types; between depressed, euthymic and manic states; and between bipolar I and II disorder. We also assessed cellular [Ca^2+^] in medication-free individuals, and addressed diagnostic specificity by examining studies which directly compared bipolar disorder with schizophrenia or unipolar depression. Finally, we reviewed studies in which other parameters of cellular Ca^2+^ had been measured.

Given these objectives, we aimed to identify all studies in which individuals with bipolar disorder were compared with healthy controls, and which measured one or more of the following parameters: intracellular Ca^2+^ concentrations, at baseline or after stimulation; measures of calcium flux or mobilisation; calcium-mediated electrophysiological signals. All cell types (including those derived from induced pluripotent stem cells [iPSC]) were included. We did not consider data which were only published in abstracts, or in non-peer reviewed publications (e.g. book chapters), or in languages other than English. We excluded studies of blood or other body fluids, and studies measuring calcium-related molecules such as calcium channels, calcium-binding proteins, Ca^2+^-ATPase, or TRPC7. We also excluded animal models of bipolar disorder.

To identify relevant studies we searched the Web of Science, All Databases, refined to the Web of Science Core Collection and Medline. The search terms were (calcium or Ca^2+^) and (‘bipolar disorder’ or ‘bipolar disease’ or ‘bipolar I’ or ‘bipolar II’ or ‘bipolar depression’ or ‘manic-depressi*’ or ‘manic depressi*’ or ‘affective’ or ‘mania’ or ‘manic’). In addition, we searched the reference list of all eligible studies, all citations of eligible studies, and our own reprint collections. The last search was on 18th July 2019.

Titles and abstracts of studies retrieved by the search strategy were screened independently by two of the authors. The full text of all potentially eligible articles (and, where relevant, their supplementary information) was also obtained and independently assessed by two authors. We resolved any ambiguities about eligibility through discussion. The extracted information comprised: year of publication; numbers of cases and controls; diagnostic criteria for bipolar disorder; subtype of bipolar disorder; cell type; mood state of cases (manic, euthymic, depressed, or not stated); medication status of cases; methodology used; Ca^2+^ parameter(s) measured; mean and standard deviation of eligible datasets. Where necessary, we converted standard errors and confidence intervals to standard deviations. Graphical data were extracted using WebPlotDigitizer version 4.1 (https://automeris.io/WebPlotDigitizer/). Two authors independently extracted data; we resolved any discrepancies by discussion with other authors. We contacted study authors where aspects of the data were unclear. Most measures of quality assessment (e.g. randomisation) do not apply to a systematic review of this kind; however we did check whether the study reported whether analyses wereconducted blind to diagnosis, and whether all subjects were accounted for in the analyses.

Where three or more studies used a comparable measure of Ca^2+^, we pooled their results using random effects meta-analysis in Cochrane Community Review Manager 5.3 (https://community.cochrane.org/help/tools-and-software/revman-5), with standardised mean differences, 95% confidence intervals and two-sided *p* values for each outcome. Where subgroups needed to be combined, we used the formula for weighted means in the Cochrane handbook (https://handbook-5-1.cochrane.org/). We assessed heterogeneity using the *I*² statistic and *χ*² test.

## Results

**Fig. 1:**
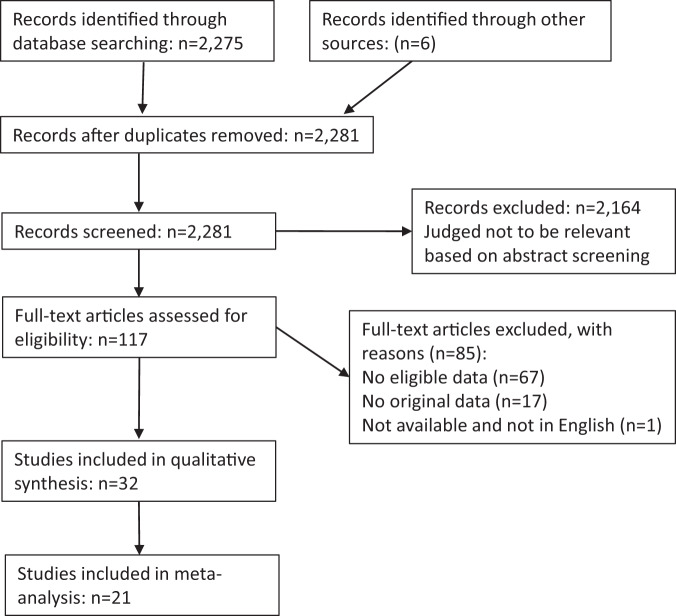
**PRISMA statement.**

A PRISMA diagram is shown (Fig. 1). We identified 2281 articles from our search strategy, and after screening 117 papers we identified 32 studies meeting our criteria for inclusion in the systematic review [2, 3, 21–50]. Eighty-five other studies were reviewed but excluded; they are listed in Supplementary Table 1, along with their reason for exclusion. These included several instances where the same data were reanalysed or subsumed in a second publication; we are grateful to authors who clarified these issues. Regarding the measurable quality indices, all subjects were accounted for in all studies, but no studies commented on blinding.

The 32 studies in the systematic review were published between 1988 and 2017. The majority used platelets (*n* = 22); lymphocytes were used in four studies, and transformed cells (B-lymphoblasts and iPSC-derived neurons) used in six. Some studies used more than one cell type. The commonest parameters measured were basal ([Ca^2+^]_b_; *n* = 22) and stimulated ([Ca^2+^]_s_; *n* = 20) free intracellular calcium ion concentration. The most widely used stimulants were 5-hydroxytryptamine (5-HT; *n* = 8) and thrombin (*n* = 7). The standard methodology for these studies was to use Fura2, a high affinity calcium indicator dye. Studies using neurons mostly measured electrophysiological rather than biochemical parameters of Ca^2+^ (*n* = 5).

Study sample sizes ranged from 2 to 215 bipolar disorder cases and 2–70 controls. Diagnoses were made using DSM-III (*n* = 3), DSM-IIIR (*n* = 12), DSM-IV (*n* = 10), other (*n* = 4), or unstated (*n* = 3) criteria. Some studies included one or more other psychiatric disorder groups, notably unipolar depression (*n* = 10) and schizophrenia (*n* = 5). Within bipolar disorder, some studies did not note (or did not separate) mood states, but the majority included one or more subgroups defined as being depressed, manic, or euthymic (in remission) at the time of sampling.

### Meta-analyses of basal cellular Ca^2+^ concentration

#### Bipolar disorder versus controls

**Fig. 2:**
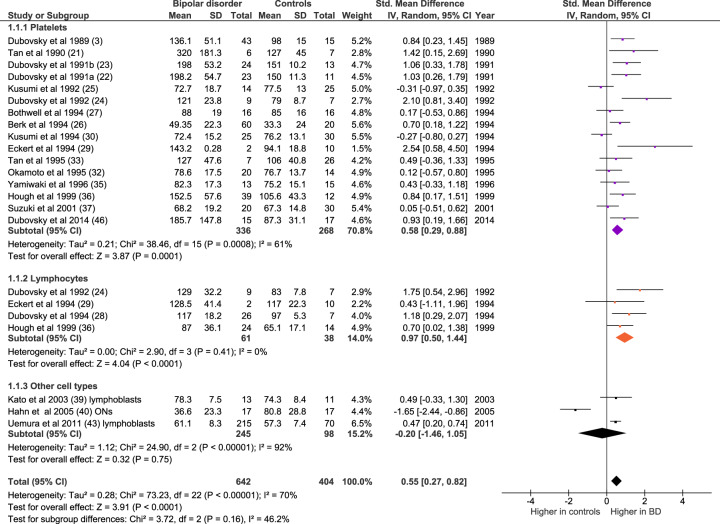
Forest plot showing basal intracellular [Ca^2+^] in bipolar disorder compared with controls, both overall and in platelet (purple), lymphocyte (orange) and other cell type (dark grey) subgroups. IV inverse variance, ONs olfactory neurons.

After exclusion of duplicate datasets, 20 eligible meta-analysable studies measuring [Ca^2+^]_b_ in bipolar disorder (all mood states and cell types combined, *n* = 642) and controls (*n* = 404) were identified (Fig. 2). Meta-analysis shows elevated [Ca^2+^]_b_ in bipolar disorder (*n* = 23 datasets; *Z* = 3.91, *p* < 0.0001; SMD 0.55, 95% confidence interval 0.27–0.82). The mean increase of [Ca^2+^]_b_ in bipolar disorder was 29% (range −54–152%).The increase is significant in studies of platelets (*n* = 16 datasets, of 336 patients and 268 controls; *Z* = 3.87, *p* < 0.0001; SMD 0.58 [0.29–0.88]) and lymphocytes (*n* = 4 datasets, of 61 patients and 38 controls; *Z* = 4.04, *Z* = 4.04, *p* < 0.0001, SMD 0.97 [0.50–1.44]); the result in the category of ‘other cells’ (B-lymphoblasts and olfactory neurons) is not significant, but there is no overall heterogeneity between cell types (*p* = 0.16).

#### Effect of mood state

**Fig. 3:**
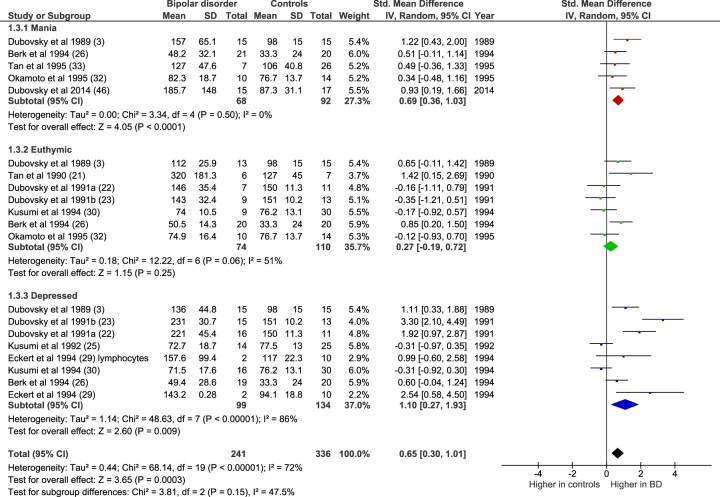
Forest plot showing basal intracellular [Ca^2+^] in mania (red), euthymia (green), and bipolar depression (blue) compared with controls. All studies are in platelets except where noted. IV inverse variance.

The majority of studies allowed separate comparisons to be made for mania, euthymia, and depression (Fig. 3). Compared with controls, [Ca^2+^]_b_ was elevated in mania (*n* = 5 datasets, of 68 patients and 92 controls; *Z* = 4.05, *p* < 0.0001; SMD 0.69 [0.36–1.03]) and in bipolar depression (*n* = 8 datasets, of 99 patients and 134 controls; *Z* = 2.60, *p* = 0.009; SMD 1.10 [0.27–1.93]). However, in remission, [Ca^2+^]_b_ did not differ from controls (*n* = 7 datasets, of 74 patients and 110 controls; *Z* = 1.15, *p* = 0.25; SMD 0.27 [−0.19–0.72]).

#### Effect of medication

**Fig. 4:**
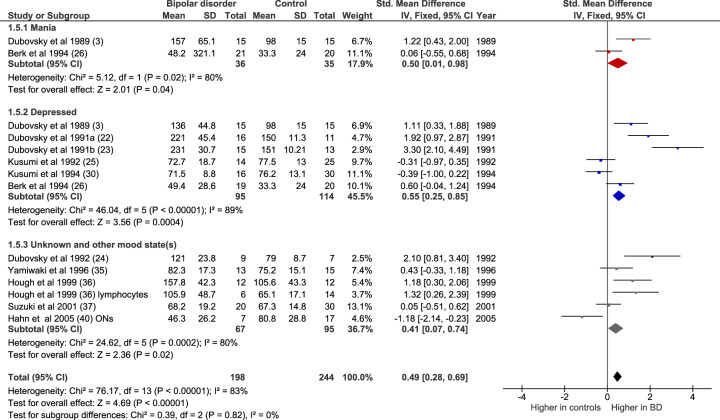
Forest plot showing basal intracellular [Ca^2+^] in unmedicated bipolar disorder, both overall and in mania (red), depressed (blue) and unknown or other mood state (grey) subgroups, compared with controls. All studies are in platelets except where noted. IV inverse variance, ONs olfactory neurons.

We also examined results of studies in which only unmedicated patients were included. In three of these studies the subjects were drug naïve [25, 26, 30]; in the others they had been drug free for between 2 weeks and 3 months. Figure 4 shows that there was an elevated [Ca^2+^]_b_ in unmedicated bipolar disorder (*n* = 14 datasets, of 198 patients and 244 controls; *Z* = 4.69, *p* < 0.00001; SMD 0.49 [0.28–0.69]). The increase was significant in mania (*n* = 2 studies, of 36 patients and 35 controls; *Z* = 2.01, *p* = 0.04, SMD 0.50 [0.01–0.98], in bipolar depression (*n* = 6 studies, of 95 patients and 114 controls; *Z* = 3.56, *p* = 0.0004; SMD 0.55 [0.25–0.85]), and in five studies, in which mood state was either unknown or different states were grouped together (*n* = 6 datasets, of 67 patients and 95 controls; *Z* = 2.36, *p* = 0.02; SMD 0.41 [0.07–0.74]). There were no meta-analysable studies of unmedicated euthymic patients as only one study included such a group [40].

#### Bipolar disorder compared with other diagnoses

**Fig. 5:**
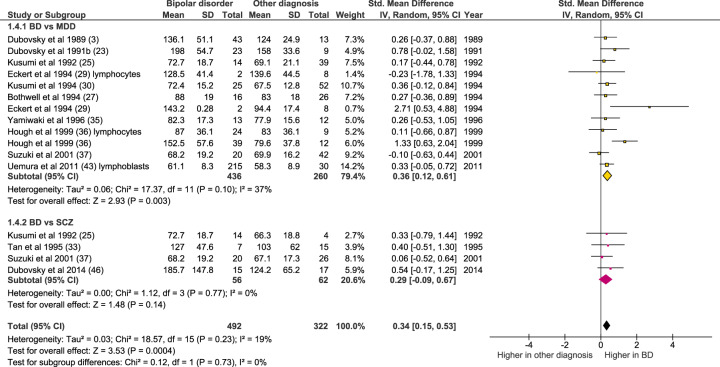
Forest plot showing basal intracellular [Ca^2+^] in bipolar disorder compared with unipolar depression (yellow) and schizophrenia (pink). All studies are in platelets except where noted. IV inverse variance.

As shown in Fig. 5, [Ca^2+^]_b_ in bipolar disorder was significantly higher than in unipolar depression (*n* = 12 datasets, of 436 bipolar disorder individuals and 260 with unipolar depression; *Z* = 2.93, *p* = 0.003; SMD 0.36 [0.12–0.61]). There was no significant difference in [Ca^2+^]_b_ between bipolar disorder and schizophrenia, albeit based on a much smaller sample size (*n* = 4 datasets, of 56 bipolar disorder individuals and 62 with schizophrenia; *Z* = 1.48, *p* = 0.14; SMD 0.29 [−0.09–0.67]).

### Meta-analyses of cellular Ca^2+^ concentration after stimulation with 5-HT or thrombin

**Fig. 6:**
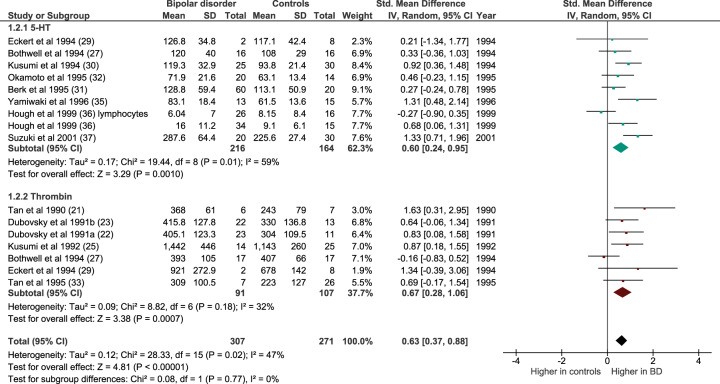
Forest plot showing intracellular [Ca^2+^] after stimulation with 5-HT (blue–green) or thrombin (dark red) in bipolar disorder compared with controls. All studies are in platelets except where noted. IV inverse variance.

Intracellular [Ca^2+^] is regulated by a range of extrinsic stimuli. A number of studies have therefore examined [Ca^2+^]_s_ in bipolar disorder, in addition to or instead of [Ca^2+^]_b_. The most commonly used stimulants are 5-HT and thrombin. Both were amenable to meta-analysis, which showed an enhanced response in bipolar disorder (*n* = 16 datasets, of 307 patients and 271 controls; *Z* = 4.81, *p* < 0.0001; SMD 0.63 [0.28–1.06]; Fig. 6). The mean increase of [Ca^2+^]_s_ in bipolar disorder was 25.2% (range −25.9–75.7%). The effect was significant for 5-HT (*n* = 9 datasets, of 216 patients and 164 controls; *Z* = 3.29, *p* < 0.001; SMD 0.60 [0.24–0.95]) and for thrombin (*n* = 7 datasets, of 91 patients and 107 controls; *Z* = 3.38, *p* = 0.0007; SMD 0.67 [0.28–1.06]).

Both stimulants were used across a range of doses, and in some studies the dose was unclear. The significantly greater responses in bipolar disorder than controls remained if analyses were restricted to the commonest concentration used in these studies (10 μM 5-HT; *Z* = 5.78, *p* < 0.00001; SMD 0.90 [0.59–1.20]) and thrombin (0.1 U/ml; *Z* = 3.79, *p* = 0.0001; SMD 0.81 [0.39–1.22]). Two studies used more than one concentration of 5-HT: Berk et al. (ref. 31) used 100 nM, 500 nM and 1 μM, and Kusumi et al. (ref. 30) used 300 nmol and 10 μmol. In both instances, the enhanced [Ca^2+^]_s_ response in bipolar disorder was of similar magnitude at each concentration tested.

### Investigation of heterogeneity and bias

As shown in Figs. 2–6, many of the meta-analyses showed significant heterogeneity (*I*^2^ > 50% or chi square *p* < 0.05). We could not find any overall explanations for this, although Hahn et al. [40] was an outlier in Figs. 2 and 6. Omission of this study reduced *I*^2^ to below 50%. It may be relevant that Hahn et al. [40] was the only meta-analysed study to use olfactory neurons. Inspection of funnel plots did not show any clear evidence of publication bias (for funnel plots and comments, see Supplementary Fig. 1).

The original study of [Ca^2+^]_b_ in bipolar disorder suggested greater inter-subject variability in cases than in controls [3]. To investigate this, we calculated variability (standard deviation divided by the mean) for each study included in Figs. 2 and 3. There was a trend towards greater variability in bipolar disorder (0.31 ± 0.18 vs. 0.22 ± 0.15, *p* = 0.09, *t*-test), which became nominally significant if one study with an outlying value in the control group [26] was excluded (0.31 ± 0.18 vs. 0.20 ± 0.11, *p* = 0.02).

### Qualitative syntheses

Not all data met our criteria for meta-analysis (usually because less than three studies had measured the same parameter). The main findings of these studies are summarised here.

We had intended to compare bipolar I with bipolar II disorder, but only two studies presented results in this way. Hough et al. [36] found no differences in [Ca^2+^]_b_ or [Ca^2+^]_s_ between these subgroups in platelets or lymphocytes, whereas Uemura et al. [43] in a large sample of B-lymphoblasts, found [Ca^2+^]_b_ elevated in bipolar I but not bipolar II disorder.

Two studies measured parameters related to [Ca^2+^]_b_ but using different methods from the standard Fura2 technique. They did not find significant group differences [2, 38]. Berk et al. [34] measured ^45^Ca^2+^ uptake into platelets, and found no differences between bipolar disorder subjects and controls.

In addition to the 5-HT and thrombin data summarised in Fig. 6, several other stimulants have been studied for their effect on [Ca^2+^]_s_. These include dopamine [26], platelet activating factor [27], phytohaemagglutinin [29], levetiracetam [47], odorants [40], and thapsigargin [41, 42]. Combinations of these and other stimulants have also been applied [3, 28, 36, 39, 46]. No consistent pattern of results has emerged, with many of the studies reporting no significant differences between bipolar disorder and control individuals.

Recent studies have begun to use iPSC-derived neuronal cells to examine Ca^2+^ functioning in bipolar disorder, usually as a small part of investigations focusing on other parameters and on lithium responsiveness of the cells [45, 48, 50]. Chen et al. [45] pretreated induced neurons with lithium and showed a greater reduction in calcium transient and wave amplitude in neurons from individuals with bipolar disorder than from controls. Mertens et al. [48] reported a greater frequency of calcium events in iPSC-derived neurons from bipolar disorder individuals than from controls, and Tobe et al. [50] found altered kinetics of calcium transients in iPSC-derived neurons from bipolar subjects who had responded to lithium, but not in lithium non-responders, compared with healthy controls. Whilst all three studies contain some support for differential cellular Ca^2+^ signalling in bipolar disorder, all were based on very small samples, and no specific finding has yet been unequivocally replicated. Completing the results in neuronal-like cells, no differences have been observed in L-type calcium current properties in preliminary studies of olfactory neuronal cells from individuals with bipolar disorder compared with controls [44, 49].

## Discussion

Calcium signalling has been the most studied in vitro parameter in bipolar disorder [51], resulting in 32 studies eligible for our systematic review and 21 available for meta-analysis. Our main finding is that there is robust evidence that intracellular [Ca^2+^] is elevated in bipolar disorder (Figs. 2–6). This is seen in unmedicated patients, is present in platelets and lymphocytes, occurs in mania and depression, and under basal conditions and in response to stimuli. The findings thus provide strong support for altered calcium functioning in bipolar disorder.

Before proceeding, several potential confounders should be considered. Intracellular [Ca^2+^] can be modestly affected by a range of demographic factors including age, gender, ethnicity, genetic factors, and possibly by blood pressure [52–55]. Whilst most studies have matched case and control groups by age and gender, the other factors have rarely if ever been mentioned or controlled for. The most relevant of these potential confounders is blood pressure, since there is a higher rate of cardiovascular disease and hypertension in patients with bipolar disorder [56, 57]. However, it seems unlikely that this could explain more than a small fraction of the diagnostic differences, especially since a relationship between blood pressure and intracellular [Ca^2+^] has not been consistently observed [52, 54, 55]. Nevertheless, future studies will benefit from a more careful assessment of, and control for, blood pressure and other cardiovascular and metabolic indices.

The fact that the differences were seen in unmedicated patients, and in lymphoblasts, in which any residual drug effects would likely be removed, argues against the elevated [Ca^2+^] in bipolar disorder being attributable to lithium or other psychotropic drugs. A full discussion of whether bipolar disorder medications normalise cellular [Ca^2+^] is beyond the scope of this systematic review. However, there is considerable evidence that this may be the case for lithium [3, 18, 20, 58], with weaker evidence for other mood stabilisers [59]. The question of how medication impacts on [Ca^2+^] is relevant to the finding that [Ca^2+^]_b_ was not significantly altered in bipolar disorder patients in remission (Fig. 4). It is possible that altered [Ca^2+^]_b_ is only seen during a mood episode; alternatively, it may be a trait which is present throughout the illness but which is normalised by medication (since all euthymic patients were on medication). It will be valuable in future studies to include euthymic patients who are medication free to address this issue. It will be of even more interest to study [Ca^2+^]_b_ (and [Ca^2+^]_s_) longitudinally, including in high-risk subjects, to determine the temporal trajectory of elevated [Ca^2+^] across the course of bipolar disorder.

Cellular [Ca^2+^] has been measured in other disorders, notably unipolar (major) depression and in schizophrenia. Studies which also included a bipolar disorder group were included in a meta-analysis (Fig. 5), revealing that [Ca^2+^]_b_ is lower (or increased less) in unipolar depression than in bipolar disorder. Bipolar disorder did not differ significantly from schizophrenia, albeit based on a much smaller dataset and with a consequent lack of power for this comparison. Regarding the overlap with schizophrenia, it is notable that there is some evidence that the elevation of [Ca^2+^]_b_ is seen in bipolar I rather than bipolar II, at least in lymphoblasts [43]. There were insufficient data to review systematically the diagnostic specificity of [Ca^2+^]_s_, but one study suggested a greater response in bipolar disorder than in major depression [60]. Overall, the data overall suggest that raised intracellular [Ca^2+^] is prominent in, but probably not restricted to, bipolar disorder.

Intracellular [Ca^2+^] is subject to intricate and complex regulation [61–63], and various explanations have been proffered for the increased [Ca^2+^] seen in bipolar disorder [18–20], including whether the primary cause is excess influx, decreased efflux, or altered compartmentalisation of [Ca^2+^] within the cell. The latter view links [Ca^2+^] to mitochondrial and endoplasmic reticulum theories of the disorder, since these organelles are critical to regulation of intracellular [Ca^2+^] [19, 64]. Altered expression or function of molecules involved in Ca^2+^ fluxes and Ca^2+^ buffering in bipolar disorder has also been reported (e.g. refs. [65–67] and see Supplementary Table 1). The results of this systematic review do not bear directly upon these issues, but do provide a strong rationale to investigate further the explanation for the abnormality. One point to emphasise that the meta-analysed studies only measure free intracellular Ca^2+^ [68]. This is a tiny fraction (<1%) of total cellular [Ca^2+^], with the vast majority being sequestered in the organelles mentioned and in membrane-associated microdomains. As such, the measured parameter is the tip of the cellular [Ca^2+^] iceberg, and hence the 25–30% elevations seen in bipolar disorder could result from a small difference in one or more of the intracellular calcium stores and the molecules which regulate them. That is, the core cellular ‘calcium abnormality’ of bipolar disorder could be extremely subtle. It remains to be determined whether the elevation in free intracellular [Ca^2+^] in bipolar disorder is itself deleterious, or simply a marker of the underlying disturbance.

The recent resurgence of interest in calcium in bipolar disorder has been fostered by the genomic data showing that VGCCs are part of the genetic risk architecture, implicating a role for Ca^2+^ signalling in the disorder [12, 16, 17]. The question arises as to whether the genetic findings are directly related to the elevated intracellular [Ca^2+^] observed in platelets and lymphocytes. At first sight this seems unlikely since VGCCs have been considered to be signature channels of cellular excitability [69] (i.e. limited to neurons and myocytes), with [Ca^2+^] in non-excitable cells being controlled by other mechanisms such as store-operated Ca^2+^-entry. However, there is increasing evidence that VGCCs are in fact expressed and functional in many non-excitable cells [69], including in some lymphocytes [70, 71] and in glia [72–74]. It therefore cannot be excluded that VGCCs have a role in [Ca^2+^] regulation in many cell types, although this would seem likely to be at most a minor contribution to the results reported here, especially since VGCCs are not expressed in platelets or their megakaryocyte precursors [75]. Finally, it would be of interest to study intracellular [Ca^2+^] in glia in bipolar disorder, since these cells are implicated in its pathophysiology [76, 77], and Ca^2+^ dysregulation in the disorder may involve these cells as well as the presumed primary neuronal locus of dysfunction. Despite the uncertainties as to its cause and molecular basis, the increase in intracellular [Ca^2+^] found in bipolar disorder is broadly supportive of the possibility that novel calcium channel antagonists, or related drugs acting upon calcium signalling, could be of potential therapeutic value, especially if designed to be brain selective [78–80].

## Supplementary information

Supplementary Table 1

Supplementary Figure 1
