# Supplementary material for: Cellular calcium in bipolar disorder: systematic review and meta-analysis
Source: Mol Psychiatry. 2019 Dec 4;26(8):4106–16. doi: 10.1038/s41380-019-0622-y (PMC8550977; doi:10.1038/s41380-019-0622-y)
Supplement: Supplementary file 1 — Supplementary Table 1 [file 41380_2019_622_MOESM1_ESM.docx]

**Supplementary Table 1. List of studies excluded from systematic review and reason for their exclusion**

| **Study** | **Year** | **Reason for exclusion** | **Further details** |
| --- | --- | --- | --- |
| Aillon | 1971 | Review. No original data. |  |
| Albert et al | 2015 | No eligible data. | No control group - followed up Li+ treated patients |
| Alexander et al | 1986 | No eligible data. | Measured Ca^2+^ ATPase activity |
| Aronoff et al | 1971 | No eligible data. | No control group - followed up Li+ treated patients |
| Baastrup et al | 1978 | No eligible data. | Serum calcium (and PTH) |
| Bech et al | 1978 | No eligible data. | CSF and blood |
| Berk and Kirchmann | 1995 | Basal Ca uptake data in Berk et al 1996 | ^45^Ca^2+^ uptake, after verapamil or flunarizine |
| Bunney and Murphy | 1976 | Review. No original data. |  |
| Carman and Wyatt | 1979a | No eligible data. |  |
| Carman and Wyatt | 1979b | No eligible data. |  |
| Carman and Wyatt | 1979c | No eligible data. |  |
| Carman et al | 1977 | No eligible data. |  |
| Carman et al | 1979 | No eligible data. | No control group; serum calcium at different phases of illness |
| Choi et al | 1981 | No eligible data. | No control group. Ca2+ ATPase in response to lithium in BD. |
| Christiansen et al | 1975 | No eligible data. | Serum calcium |
| Christiansen et al | 1976 | No eligible data. | Serum calcium |
| Coppen et al | 1966 | No eligible data. |  |
| Coppen | 1967 | Review. No original data. |  |
| Corson et al | 2001 | Data subsumed in Uemura et al 2011 |  |
| Costa et al | 1982 | No eligible data | Measure of dense-body calcium |
| Dubovsky | 1993 | Review. No original data. |  |
| Dubovsky and Franks | 1983 | Review. No original data. |  |
| Dubovsky et al | 1992 | Review. No original data. |  |
| El-Mallakh and Jaziri | 1990 | Review. No original data. |  |
| Emamghoreishi et al | 1997 | Data subsumed in Uemura et al 2011 |  |
| Emamghoreishi et al | 2000 | Same Ca2+ data as in Emamghoreishi et al 1997 |  |
| Flach | 1964 | No eligible data. | No control group; data in bipolar patients not separable from other diagnoses |
| Förstner et al | 1994 | No eligible data. | Data in bipolar patients not separable from other diagnoses |
| Franks et al | 1982 | No eligible data. | Serum calcium |
| Geetha et al | 1983 | No eligible data. | Serum calcium |
| Gerner et al | 1977 | No eligible data. |  |
| Gerner et al | 1979 | No eligible data. | No bipolar subjects; serum calcium |
| Gerner et al | 1984 | No eligible data. |  |
| Gershon | 1970 | Review. No original data. |  |
| Glen | 1985 | Review. No original data. |  |
| Hayashi et al | 2015 | No eligible data. | Expression of Ca2+-related molecules |
| Herman | 1981 | No eligible data. | Case report |
| Hesketh et al | 1977 | No eligible data. | ATPase activities |
| Hudson et al | 1993 | Review. No original data. |  |
| Hullin et al | 1975 | No eligible data. |  |
| Jimerson et al | 1979 | No eligible data. | CSF Ca2+ |
| Joborn et al | 1988 | No eligible data. | Study of hyperparathyroidism |
| Jope et al | 1996 | No eligible data. | Ca2+ stimulated PLC activity in brain |
| Kassir and Meltzer | 1991 | No eligible data. | Ca2+ inhibition of NaKATPase |
| Koenig and Jope | 1988 | No eligible data. | Rat study |
| Komatsu et al | 1995 | No eligible data. | Serum calcium |
| Kusumi et al | 2000 | No eligible daa | Data included in Kusumi et al 1994 |
| Li and E-Mallakh | 2004 | No eligible data | Calcium responses to Na+ pump inhibition |
| Linder et al | 1989 | No eligible data. | Serum calcium |
| Linnoila et al | 1983 | No eligible data. | ATPase, and 2 BD patients inseparable from MDD patients |
| Macdonald et al | 1984 | No eligible data. | Calmodulin stimulated Ca2+ATPase |
| Machado-Vieira et al | 2011 | No eligible data. | No control group. Bcsl-2 genotype comparisons within BD. |
| Marel et al | 1982 | No eligible data. |  |
| Mellerup and Mellerup | 1984 | No eligible data. | Urinary calcium |
| Mellerup et al | 1974 | Review. No original data. |  |
| Mellerup et al | 1976 | No eligible data. | Serum calcium |
| Mellerup et al | 1979 | No eligible data. |  |
| Meltzer | 1986 | Review. No original data. |  |
| Meltzer and Kassir | 1983 | No eligible data. | Calmodulin stimulated Ca2+ATPase |
| Meltzer et al | 1988 | No eligible data. | Calmodulin stimulated Ca2+ATPase |
| Moutsatsou et al | 2014 | No eligible data. | PDF requested from author 23.10.18, received 29.10.18. |
| Naylor et al | 1972 | No eligible data. | Serum calcium |
| Nielsen et al | 1977 | No eligible data. | Renal calcium excretion |
| Pavlinac et al | 1979 | No eligible data. | Rat study |
| Perova et al | 2010 | No eligible data | Responses to mood stabilisers; no new BD vs con data |
| Plenge and Rafaelsen | 1982 | No eligible data. | Serum and renal calcium |
| Post et al | 1980 | No eligible data. | CSF calcium |
| Roedding et al | 2012 | No eligible data | Eligible BD vs control data already reported in earlier publications |
| Soares and Mallinger | 1997 | Review. No original data. |  |
| Stern et al | 1996 | No eligible data. | Serum and urinary calcium |
| Suzuki et al | 2003 | Controls chosen because they had previously shown high [Ca2+] |  |
| Tandon et al | 1988 | No eligible data. | CSF calcium |
| Tupin et al | 1968 | No eligible data. | Lithium effects on electrolytes |
| Uemura et al | 2016 | No eligible data | BD vs control data already reported in Emamghoreishi et al 1997 and Uemura et al 2011 |
| van Calker et al | 1993 | No eligible data | Can't separate unipolar from bipolar subjects |
| Viswanath et al | 2015 | Review. No original data. |  |
| Walden et al | 1992 | Review. No original data. | And in German |
| Wasserman et al | 2004 | No eligible data | Responses to mood stabilisers; no new BD vs con data |
| Wood | 1985 | Review. No original data. |  |
| Xu et al | 2006 | No eligible data | Eligible BD vs control data reported in Uemura et al 2011 |
| Yamiwaki et al | 1998 | Review. No original data. |  |
| Yoon et al | 2001a | No eligible data. | Ca indices taken from Emamghoreishi et al 1997 |
| Yoon et al | 2001b | No eligibe data | Ca indices taken from Emamghoreishi et al 1997 |
| Yoshimizu et al | 2015 | No eligible data. | Genotype comparisons, no BD vs con data |
| Zapletalek and Groh | 1978 | Not available | Journal not accessible; article not in English |

**References to studies excluded from systematic review**

Aillon GA. Biochemistry of affective disorders. Psychosomatics. 1971; 12(4): 260-72.

Albert U, De Cori D, Aguglia A, Barbaro F, Lanfranco F, Bogetto F, et al. Effects of maintenance lithium treatment on serum parathyroid hormone and calcium levels: a retrospective longitudinal naturalistic study. Neuropsychiatr Dis Treat. 2015; 11: 1785-91.

Alexander DR, Deeb M, Bitar F, Antun F. Sodium-potassium, magnesium, and calcium ATPase activities in erythrocyte membranes from manic-depressive patients responding to lithium. Biol Psychiatry. 1986; 21(11): 997-1007.

Aronoff MS, Evens RG, Durell J. Effect of lithium salts on electrolyte metabolism. J Psychiatr Res. 1971; 8(2): 139-59.

Baastrup PC, Christiansen C, Transbol I. Calcium metabolism in lithium-treated patients. Relation to uni-bipolar dichotomy. Acta Psychiatr Scand. 1978; 57(2): 124-8.

Bech P, Kirkegaard P, Bock E, Johannesen M, Rafaelsen OJ. Hormones, electrolytes, and cerebrospinal fluid proteins in manic-melancholic patients. Neuropsychobiology. 1978; 4: 99-112.

Berk M, Kirchmann NH. Enhanced blockade of 45Ca2+ uptake into platelets in manic patients with bipolar affective disorder with flunarizine and verapamil. . Human Psychopharmacology. 1995; 10: 299-303.

Bunney WE, Jr., Murphy DL. Neurobiological Considerations of the mode of action of lithium carbonate in the treatment of affective disorders? Pharmakopsychiatr Neuropsychopharmakol. 1976; 9(3): 142-7.

Carman JS, et al. Calcium and electroconvulsive therapy of severe depressive illness. Biol. Psychiatry 1977; 12 (1): 5-17.

Carman JS, Post RM, Runkle DC, Bunney WE, Jr., Wyatt RJ. Increased serum calcium and phosphorus with the 'switch' into manic or excited psychotic state. Br J Psychiatry. 1979; 135: 55-61.

Carman JS, Wyatt RJ. Calcium: pacesetting the periodic psychoses. Am J Psychiatry. 1979a; 136(8): 1035-9.

Carman JS, Wyatt RJ. Calcium: bivalent cation in the bivalent psychoses. Biol Psychiatry. 1979b; 14(2): 295-336.

Carman JS, Wyatt RJ. Use of calcitonin in psychotic agitation or mania. Arch Gen Psychiatry. 1979c; 36(1): 72-5.

Choi SJ, Derman RM, Lee KS. Bipolar affective disorder, lithium carbonate and Ca++ ATPase. Journal of Affective Disorders. 1981; 3: 77-9.

Christiansen C, Baastrup PC, Transbol I. Osteopenia and dysregulation of divalent cations in lithium-treated patients. Neuropsychobiology. 1975; 1: 344-54.

Christiansen C, Baastrup PC, Transbol I. Lithium, hypercalcaemia, hypermagnesaemia, and hyperparathyroidism. Lancet. 1976; ii: 969.

Coppen A. The biochemistry of affective disorders. Br J Psychiatry. 1967; 113(504): 1237-64.

Coppen A, Shaw DM, Malleson A, Costain R. Mineral metabolism in mania. Br Med J. 1966; 1(5479): 71-5.

Corson TW, Li PP, Kennedy JL, Macciardi F, Cooke RG, Parikh SV, et al. Association analysis of G-protein beta 3 subunit gene with altered Ca(2+) homeostasis in bipolar disorder. Mol Psychiatry. 2001; 6(2): 125-6.

Costa JL, Fay DD, Nurnberger JI, Murphy DL. Preferential accumulation of lithium in the dense bodies of human platelets. Biochem Pharmacol. 1982; 31(20): 3215-8.

Dubovsky SL. Calcium antagonists in manic-depressive illness. Neuropsychobiology. 1993; 27(3): 184-92.

Dubovsky SL, Franks RD. Intracellular calcium ions in affective disorders: a review and an hypothesis. Biol Psychiatry. 1983; 18(7): 781-97.

Dubovsky SL, Murphy J, Christiano J, Lee C. The calcium second messenger system in bipolar disorders: data supporting new research directions. J Neuropsychiatry Clin Neurosci. 1992; 4(1): 3-14.

el-Mallakh RS, Jaziri WA. Calcium channel blockers in affective illness: role of sodium-calcium exchange. J Clin Psychopharmacol. 1990; 10(3): 203-6.

Emamghoreishi M, Li PP, Schlichter L, Parikh SV, Cooke R, Warsh JJ. Associated disturbances in calcium homeostasis and G protein-mediated cAMP signaling in bipolar I disorder. Biol Psychiatry. 2000; 48(7): 665-73.

Emamghoreishi M, Schlichter L, Li PP, Parikh S, Sen J, Kamble A, et al. High intracellular calcium concentrations in transformed lymphoblasts from subjects with bipolar I disorder. Am J Psychiatry. 1997; 154(7): 976-82.

Flach FF. Calcium Metabolism in States of Depression. Br J Psychiatry. 1964; 110: 588-93.

Förstner U, Bohus M, Gebicke-Härter PJ, Baumer B, Berger M, van Calker D. Decreased agonist-stimulated Ca2+ response in neutrophils from patients under chronic lithium therapy. Eur Arch Psychiatry Clin Neurosci 243: 40-3.

Franks RD, Dubovsky SL, Lifshitz M, Coen P, Subryan V, Walker SH. Long-term lithium carbonate therapy causes hyperparathyroidism. Arch Gen Psychiatry. 1982; 39(9): 1074-7.

Geetha PR, Channabasavanna SM, Rama Rao BS. Effect of lithium on serum electrolytes. Indian J Psychiatry. 1983; 25(1): 67-9.

Gerner RH, Fairbanks L, Anderson GM, Young JG, Scheinin M, Linnoila M, et al. CSF neurochemistry in depressed, manic, and schizophrenic patients compared with that of normal controls. Am J Psychiatry. 1984; 141(12): 1533-40.

Gerner RH, Post RM, Gillin JC, Bunney WE. Biological and behavioral effects of one night's sleep deprivation in depressed patients and normals. Journal of Psychiatric Research. 1979; 15: 21-40.

Gerner RH, Post RM, Spiegel AM, Murphy DL. Effects of parathormone and lithium treatment on calcium and mood in depressed patients. Biol Psychiatry. 1977; 12(1): 145-51.

Gershon S. Lithium in mania. Clin Pharmacol Ther. 1970; 11(2): 168-87.

Glen AI. Lithium prophylaxis of recurrent affective disorders. J Affect Disord. 1985; 8(3): 259-65.

Hayashi A, Le Gal K, Sodersten K, Vizlin-Hodzic D, Agren H, Funa K. Calcium-dependent intracellular signal pathways in primary cultured adipocytes and ANK3 gene variation in patients with bipolar disorder and healthy controls. Mol Psychiatry. 2015; 20(8): 931-40.

Herman SP. Lithium, hypercalcemia, and hyperparathyroidism. Biol Psychiatry. 1981; 16(6): 593-5.

Hesketh JE, Glen AI, Reading HW. Membrane ATPase activities in depressive illness. J Neurochem. 1977; 28(6): 1401-2.

Hudson CJ, Young LT, Li PP, Warsh JJ. CNS signal transduction in the pathophysiology and pharmacotherapy of affective disorders and schizophrenia. Synapse. 1993; 13(3): 278-93.

Hullin RP, McDonald R, Allsopp MN. Further report on prophylatic lithium in recurrent affective disorders. Br J Psychiatry. 1975; 126: 281-4.

Jimerson DC, Post RM, Carman JS, van Kammen DP, Wood JH, Goodwin FK, et al. CSF calcium: clinical correlates in affective illness and schizophrenia. Biol Psychiatry. 1979; 14(1): 37-51.

Joborn C, Hetta J, Johansson H, Rastad J, Agren H, Akerstrom G, et al. Psychiatric morbidity in primary hyperparathyroidism. World J Surg. 1988; 12(4): 476-81.

Jope RS, Song L, Li PP, Young LT, Kish SJ, Pacheco MA, et al. The phosphoinositide signal transduction system is impaired in bipolar affective disorder brain. J Neurochem. 1996; 66(6): 2402-9.

Kassir S, Meltzer HL. Abnormal sensitivity of erythrocyte membrane Na/K ATPase of bipolar subjects to inhibition by calmodulin and calcium. Biol Psychiatry. 1991; 30(6): 631-4.

Koenig ML, Jope RS. Effects of lithium on synaptosomal Ca2+ fluxes. Psychopharmacology (Berl). 1988; 96(2): 267-72.

Komatsu M, Shimizu H, Tsuruta T, Kato M, Fushimi T, Inoue K, et al. Effect of lithium on serum calcium level and parathyroid function in manic-depressive patients. Endocr J. 1995; 42(5): 691-5.

Li R, El-Mallakh RS. Differential response of bipolar and normal control lymphoblastoid cell sodium pump to ethacrynic acid. J Affect Disord. 2004; 80(1): 11-7.

Linder J, Brismar K, Beck-Friis J, Saaf J, Wetterberg L. Calcium and magnesium concentrations in affective disorder: difference between plasma and serum in relation to symptoms. Acta Psychiatr Scand. 1989; 80(6): 527-37.

Linnoila M, MacDonald E, Reinila M, Leroy A, Rubinow DR, Goodwin FK. RBC membrane adenosine triphosphatase activities in patients with major affective disorders. Arch Gen Psychiatry. 1983; 40(9): 1021-6.

MacDonald E, Rubinow D, Linnoila M. Sensitivity of RBC membrane Ca2+-adenosine triphosphatase to calmodulin stimulation. Variations in patients with bipolar affective disorders. Arch Gen Psychiatry. 1984; 41(5): 487-93.

Machado-Vieira R, Pivovarova NB, Stanika RI, Yuan P, Wang Y, Zhou R, et al. The Bcl-2 gene polymorphism rs956572AA increases inositol 1,4,5-trisphosphate receptor-mediated endoplasmic reticulum calcium release in subjects with bipolar disorder. Biol Psychiatry. 2011; 69(4): 344-52.

Marel G, Frame B, Parfitt AM. Lithium and calcium metabolism. Am J Psychiatry. 1982; 139(2): 255-6.

Mellerup B, Mellerup ET. Seasonal variation in urinary excretion of calcium, magnesium and phosphate in manic-melancholic patients. Chronobiol Int. 1984; 1(1): 81-6.

Mellerup ET, Bech P, Sorensen T, Frederiksen AF, Rafaelsen OJ. Calcium and electroconvulsive therapy of depressed patients. Biol Psychiatry. 1979; 14(4): 711-4.

Mellerup ET, Lauritsen B, Dam H, Rafaelson OJ. Lithium effects on diurnal rhythm of calcium, magnesium, and phosphate metabolism in manic-melancholic disorder. Acta Psychiatr Scand. 1976; 53(5): 360-70.

Mellerup ET, Rafaelsen OJ. Heterogeneity and biochemical findings in manic-melancholic disorders. Acta Psychiatr Scand. 1974; 50(1): 104-11.

Meltzer HL. Lithium mechanisms in bipolar illness and altered intracellular calcium functions. Biol Psychiatry. 1986; 21(5-6): 492-510.

Meltzer HL, Kassir S. Abnormal calmodulin-activated calcium ATPase in manic depressive subjects. J Psychiatr Res 1983; 17 (1): 29-35.

Meltzer HL, Kassir S, Goodnick PJ, Fieve RR, Chrisomalis L, Feliciano M, et al. Calmodulin-activated calcium ATPase in bipolar illness. Neuropsychobiology. 1988; 20(4): 169-73.

Moutsatsou P, Tsoporis JN, Salpeas V, Bei E, Alevizos B, Anagnostara C, et al. Peripheral blood lymphocytes from patients with bipolar disorder demonstrate apoptosis and differential regulation of advanced glycation end products and S100B. Clin Chem Lab Med. 2014; 52(7): 999-1007.

Naylor GJ, Fleming LW, Stewart WK, McNamee HB, Le Poidevin D. Plasma magnesium and calcium levels in depressive psychosis. Br J Psychiatry. 1972; 120(559): 683-4.

Nielsen JL, Pedersen EB, Amdisen A, Darling S. Reduced renal calcium excretion during lithium therapy. Psychopharmacology (Berl). 1977; 54(1): 101-3.

Pavlinac D, Langer R, Lenhard L, Deftos L. Magnesium in affective disorders. Biol Psychiatry. 1979; 14(4): 657-61.

Perova T, Kwan M, Li PP, Warsh JJ. Differential modulation of intracellular Ca2+ responses in B lymphoblasts by mood stabilizers. Int J Neuropsychopharmacol. 2010; 13(6): 693-702.

Plenge P, Rafaelsen OJ. Lithium effects on calcium, magnesium and phosphate in man: effects on balance, bone mineral content, faecal and urinary excretion. Acta Psychiatr Scand. 1982; 66(5): 361-73.

Post RM, Ballenger JC, Hare TA, Goodwin FK, Lake CR, Jimerson DC, et al. Cerebrospinal fluid GABA in normals and patients with affective disorders. Brain Research Bulletin. 1980; 5 (suppl. 2): 755-9.

Roedding AS, Gao AF, Au-Yeung W, Scarcelli T, Li PP, Warsh JJ. Effect of oxidative stress on TRPM2 and TRPC3 channels in B lymphoblast cells in bipolar disorder. Bipolar Disord. 2012; 14(2): 151-61.

Soares JC, Mallinger AG. Intracellular phosphatidylinositol pathway abnormalities in bipolar disorder patients. Psychopharmacol Bull. 1997; 33(4): 685-91.

Stern JE, Guinjoan SM, Cardinali DP. Correlation between serum and urinary calcium levels and psychopathology in patients with affective disorders. Short communication. J Neural Transm (Vienna). 1996; 103(4): 509-13.

Suzuki K, Kusumi I, Akimoto T, Sasaki Y, Koyama T. Altered 5-HT-induced calcium response in the presence of staurosporine in blood platelets from bipolar disorder patients. Neuropsychopharmacology. 2003; 28(6): 1210-4.

Tandon R, Channabasavanna SM, Greden JF. CSF biochemical correlates of mixed affective states. Acta Psychiatr Scand. 1988; 78(3): 289-97.

Tupin JP, Schlagenhauf GK, Creson DL. Lithium effects on electrolyte excretion. Am J Psychiatry. 1968; 125(4): 536-43.

Uemura T, Green M, Warsh JJ. CACNA1C SNP rs1006737 associates with bipolar I disorder independent of the Bcl-2 SNP rs956572 variant and its associated effect on intracellular calcium homeostasis. World J Biol Psychiatry. 2016; 17(7): 525-34.

van Calker D, Forstner U, Bohus M, Gebicke-Harter P, Hecht H, Wark HJ, et al. Increased sensitivity to agonist stimulation of the Ca2+ response in neutrophils of manic-depressive patients: effect of lithium therapy. Neuropsychobiology. 1993; 27(3): 180-3.

Viswanath B, Jose SP, Squassina A, Thirthalli J, Purushottam M, Mukherjee O, et al. Cellular models to study bipolar disorder: A systematic review. J Affect Disord. 2015; 184: 36-50.

Walden J, Grunze H, Olbrich H, Berger M. [Importance of calcium ions and calcium antagonists in affective psychoses]. Fortschr Neurol Psychiatr. 1992; 60(12): 471-6.

Wasserman MJ, Corson TW, Sibony D, Cooke RG, Parikh SV, Pennefather PS, Li PP, Warsh JJ. Chronic lihtium treatment attenuates intracellular calcium mobilization. Neuropsychopharmacol. 2004; 29: 759-69.

Wood K. The neurochemistry of mania. The effect of lithium on catecholamines, indoleamines and calcium mobilization. J Affect Disord. 1985; 8(3): 215-23.

Xu C, Macciardi F, Li PP, Yoon IS, Cooke RG, Hughes B, et al. Association of the putative susceptibility gene, transient receptor potential protein melastatin type 2, with bipolar disorder. Am J Med Genet B Neuropsychiatr Genet. 2006; 141B(1): 36-43.

Yamawaki S, Kagaya A, Tawara Y, Inagaki M. Intracellular calcium signaling systems in the pathophysiology of affective disorders. Life Sci. 1998; 62(17-18): 1665-70.

Yoon IS, Li PP, Siu KP, Kennedy JL, Cooke RG, Parikh SV, et al. Altered IMPA2 gene expression and calcium homeostasis in bipolar disorder. Mol Psychiatry. 2001a; 6(6): 678-83.

Yoon IS, Li PP, Siu KP, Kennedy JL, Macciardi F, Cooke RG, et al. Altered TRPC7 gene expression in bipolar-I disorder. Biol Psychiatry. 2001b; 50(8): 620-6.

Yoshimizu T, Pan JQ, Mungenast AE, Madison JM, Su S, Ketterman J, et al. Functional implications of a psychiatric risk variant within CACNA1C in induced human neurons. Mol Psychiatry. 2015; 20(2): 162-9.

Zapletalek M, Groh J. The effect of lithium on potassium, calcium, sodium and magnesium levels in serum and erythrocytes in manic depressive psychoses. Act Nerv Super (Praha). 1978; 20(4): 295-6.
