# Supplementary material for: Cellular calcium in bipolar disorder: systematic review and meta-analysis
Source: Mol Psychiatry. 2019 Dec 4;26(8):4106–16. doi: 10.1038/s41380-019-0622-y (PMC8550977; doi:10.1038/s41380-019-0622-y)

Supplementary Figure 1. Funnel plots for each meta-analysis

Suppl. Fig. 1A: [Ca^{2+}]$_b$ in bipolar disorder compared to healthy controls (see Fig. 2 in main paper)

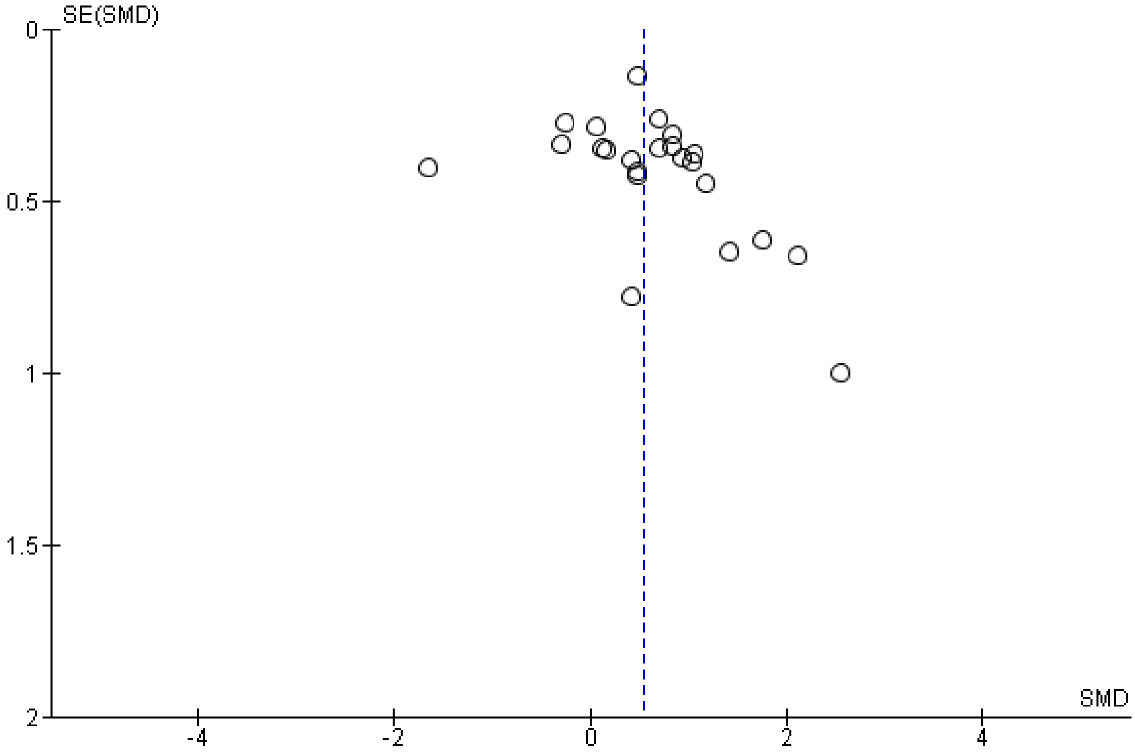

<u>Comment to Supplementary Figure 1A:</u> the funnel plot does not show any clear evidence of publication bias, however we cannot rule out the possibility of some small negative studies which are missing. To test this hypothesis, we carried out a sensitivity analysis excluding small positive studies with the largest SMD (refs. 21, 24, 29); the elevated [Ca^{2+}]$_b$ in bipolar disorder remains statistically significant (Z=2.96, p=0.003; SMD 0.45, 95% CI 0.13-0.66).

Suppl. Fig. 1B: [Ca^{2+}]$_b$ in bipolar disorder (mania, depression, and euthymia) compared to healthy controls (see Fig. 3 in main paper)

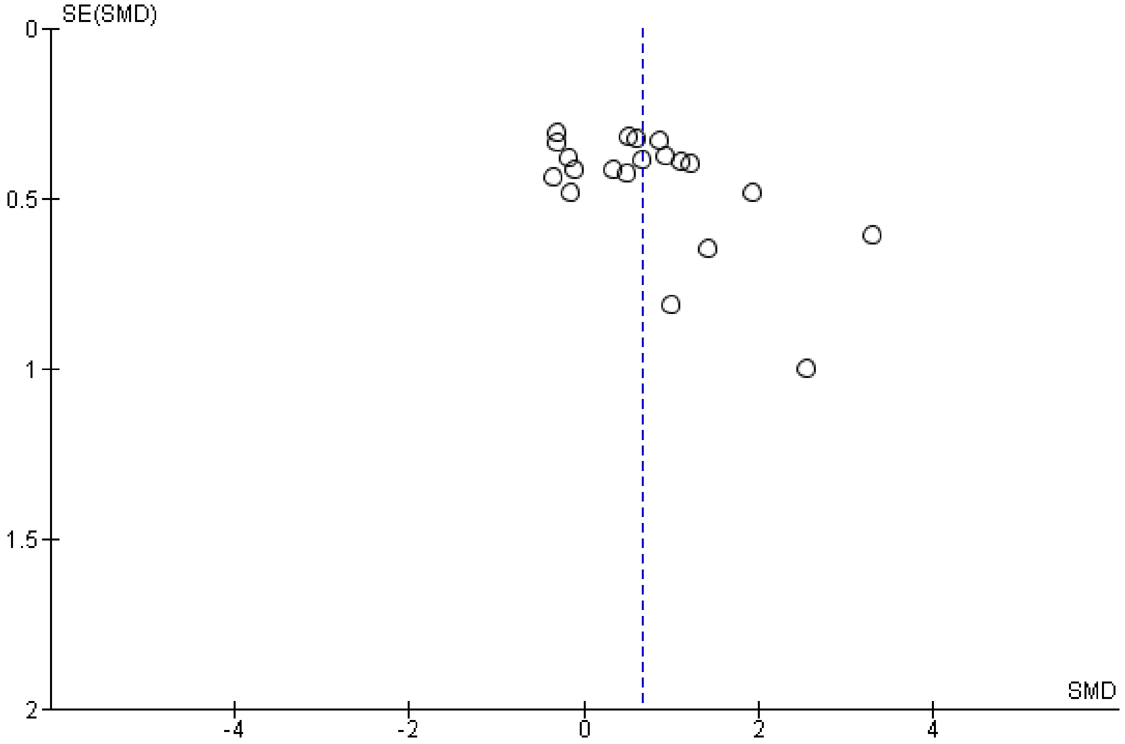

<u>Comment to Supplementary Figure 1B:</u> the funnel plot shows some evidence of an absence of small negative studies. To test this hypothesis, we carried out a sensitivity analysis excluding two small positive studies with the largest SMD (refs. 22 and 23); the elevated [Ca^{2+}]$_b$ in bipolar disorder remains statistically significant (Z=3.08, p=0.002; SMD 0.45, 95% CI 0.16-0.73).

Suppl. Fig. 1C: [Ca^{2+}]$_b$ in unmedicated bipolar disorder compared to healthy controls (see Fig. 4 in main paper).

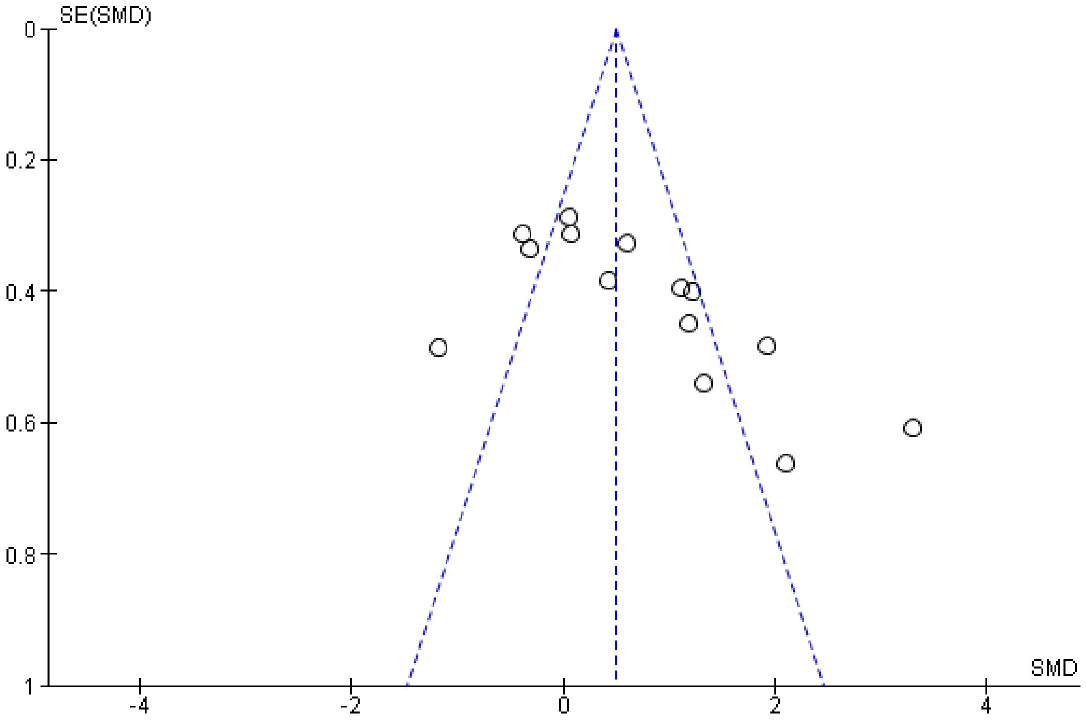

Suppl. Fig. 1D: [Ca^{2+}]$_b$ in bipolar disorder compared to major depression and schizophrenia (see Fig. 5 in main paper)

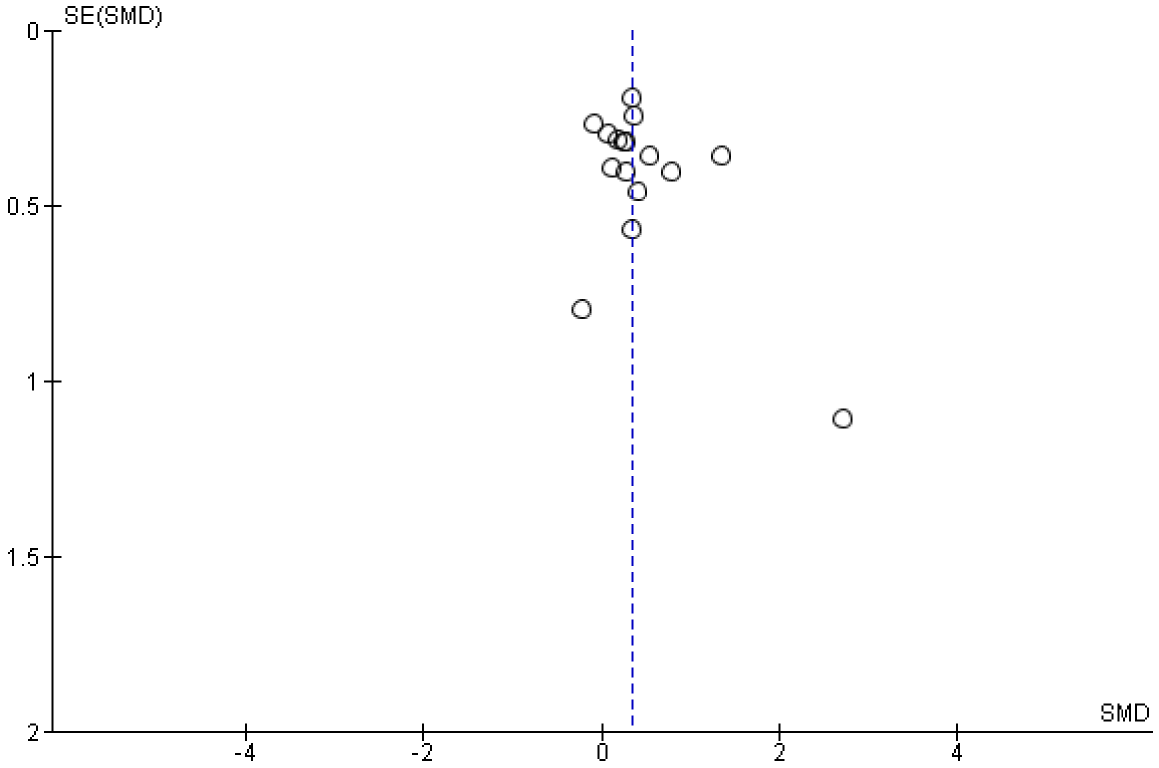

Suppl. Fig. 1E: [Ca^{2+}]$_s$ after 5-HT or thrombin stimulation in bipolar disorder compared to healthy controls (see Fig. 6 in main paper)

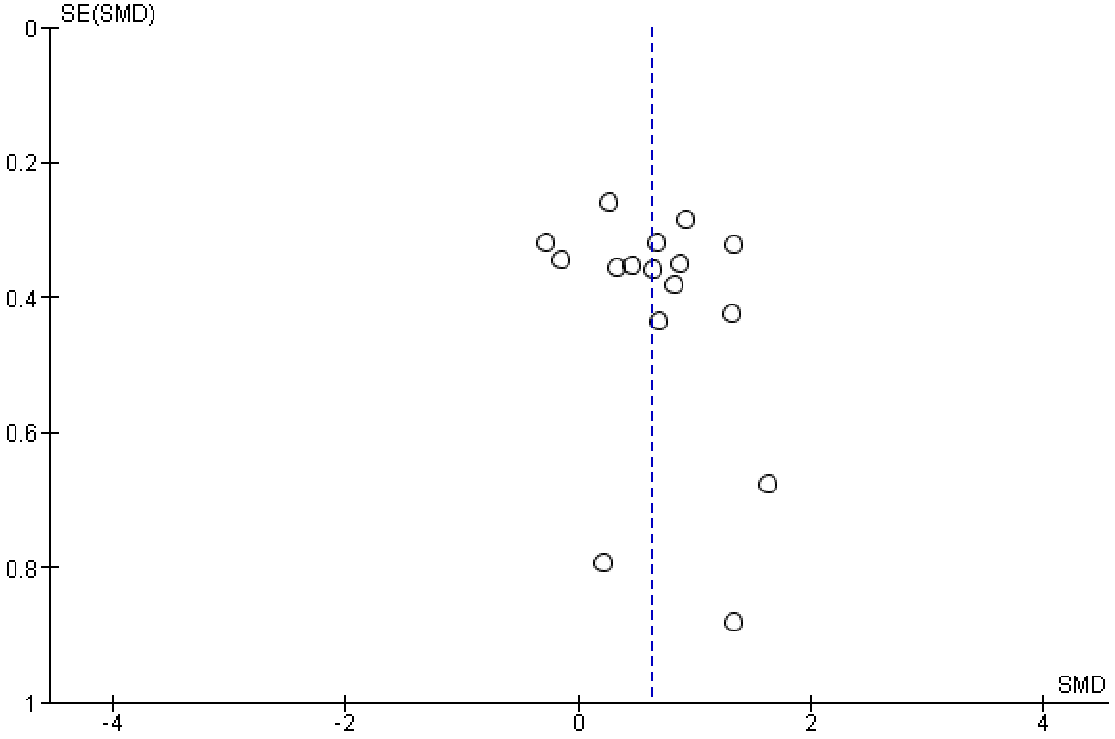

Supplement: Supplementary file 2 — Supplementary Figure 1 [file 41380_2019_622_MOESM2_ESM.pdf]
